# "The Hospital" Medical Book Supplement.—No. X

**Published:** 1908-09-19

**Authors:** 


### "THE HOSPITAL" MEDICAL BOOK SUPPLEMENT.—No. X.

CONTENTS.

**The Diseases of Children—**
The Diseases of Children ... ... ... ... ... ... ... 659
**Archives of the Middlesex Hospital—**
Archives of the Middlesex Hospital (Vols. XII. and XIII.) ... 660
**Surgery—**
A System of Syphilis ... ... ... ... ... ... ... 660
**Gynæcology and Obstetrics—**
A Manual of Midwifery ... ... ... ... ... ... ... 661
The Edinburgh Stereoscopic Atlas of Obstretrics ... ... ... 661
**Medico-Legal—**
The Law in General Medical Practice ... ... ... ... ... 661
**Climatology—**
Mediterranean Winter Resorts ... ... ... ... ... ... 662
Climate ... ... ... ... ... ... ... ... ... 662
**Miscellaneous Items—**
The Care and Nursing of the Insane ... ... ... ... ... 662
Lectures on Babies ... ... ... ... ... ... ... 662
Cancer: Relief of Pain, and Possible Cure ... ... ... ... 662

### THE DISEASES OF CHILDREN.

THE DISEASES OF CHILDREN. Edited by Drs. M. PFAUNDLER and A. SCHLOSSMANN. American translation edited by Drs. L. K. SHAW and L. LA FÉTRA. (Philadelphia and London: J. B. Lippincott Company. 1908. 4 vols. Pp. 2,154. Price £4 4s. net.)

THIS important treatise on the diseases of children is designed for the use of practising physicians. It is cyclopædic in character, the separate chapters being entrusted to different writers, both in the original German edition and in the translation. As a specimen of the publisher's art it is worthy of high praise. The volumes are large and heavy, printed on moderately glazed thick paper in clear large type, easy to read; but there are rather more printer's errors than usual in a work of this quality. The translators have carried out their duties efficiently, and the language, though occasionally involved, is not marred by Americanisms. The illustrations are admirably done, though many are unnecessary and unsuitable. The first volume is allotted to general considerations of pathogenesis, symptoms, prophylaxis, therapeutics, mortality, milk, nutrition, and metabolism. In a section of 200 pages Pfaundler gives a summary of symptoms with the underlying causes thereof. Warning is expressed against attaching undue importance to single symptoms and of the necessity of thorough clinical methods. He has provided a valuable work of reference for lecturers. We note in the chapter on general prophylaxis that desquamation is regarded as the source of infection in scarlatina and that recent observations thereon are not discussed. Pertussis also is said to be infectious through the mucus for a long time. A verbose section on general therapeutics contains a useful description of baths and spas, but makes no reference to vaccines, antitoxins, or serums. The chemistry of milk is concentrated by Raudnitz into thirteen pages of difficult reading, insufficiently clear except for the most advanced physicians. Rational common-sense methods of artificial feeding are advised, such as boiling in preference to other methods of heating milk. Goat's milk is highly recommended. Undiluted milk is not favoured and percentage methods of feeding are ignored. Artificial foods receive moderate consideration. On the whole we regard this portion as somewhat insufficient. The second volume contains chapters on diseases of the new born, puberty, general and special infections, and blood diseases. The last are inadequately discussed and differentiated. Scrofula is still thought to be a special diathesis and is described together with symptoms affecting the eye, nose, ear, skin, and joints, though it is admitted that the joint affections are tuberculous. The article on scarlet fever is alone written in the style of a clinical lecture, with many excellent illustrative cases, and an insufficient description of some complications such as otitis, joint pains, and myocarditis. Fourth disease is also honoured with a chapter, though it is regarded as rubella. Paratyphoid fever is not mentioned. The discussion on the ætiology of rheumatic fever is poor and the diplococcus rheumaticus is quite ignored. Still more curious is the mere reference to rheumatic myocarditis. In the chapters on heart disease rheumatism is stated to be comparatively infrequent in children, and therefore not a common cause of pericarditis. Hochsinger's articles on congenital heart disease and syphilis are excellent. He still believes in germinal transmission of syphilis from the father, as well as placental infection, and regards the congenital disease as not less contagious than the acquired form, though he gives little evidence in favour of his views. The other two volumes are devoted to the special systems of the body. The distressing inequality of portions of the work is shown by the limitation of tonsillar affections, pharyngitis, and adenoids to fifteen pages, whereas later on four pages are devoted to the rare and incurable tumours of the spinal cord. Retropharyngeal abscess is dismissed in a page, and the retro-œsophageal variety is not mentioned. Fischl writes discursively on diseases of nutrition, the failure of nutrition due to the unsuitable character of the food, inadequate digestion, absorption and assimilation. He is best on dyspepsia due to overfeeding, on dilatation of the colon, and on recurrent vomiting. Pfaundler's article on the pylorus is sound, full, and guarded in its conclusions. Spasm is recognised as distinct from hypertrophy. Judging from Selter's chapter on appendictis in children it must be much more common in Germany than in this country, for he states that they are seven times more frequently affected than adults. His article on hernia is decidedly sketchy. The general arrangement of respiratory diseases is unsatisfactory. Feer writes well on broncho-pneumonia, pneumonia, and empyema. He does not recognise a primary broncho-pneumonia, and recommends very vigorous treatment in pneumonic affections. Diaphragmatic pleurisy is not differentiated. The genito-urinary section is sound and practical; that on suprarenal tumours is weak. Zappert takes a wide and philosophical view of diseases of the nervous system and classifies them into endogenous, due to the abiotrophy of Gowers, and exogenous groups. Chorea is classified as a functional nervous disease, and even more unusual is the inclusion of myositis under organic nervous disease. Perhaps too much has been said about the defects of this work. It is disappointing in that it is not as complete as its size would suggest. One wishes for chapters on anæsthesia, common affections of the eyes and ear, constipation in babies, and fuller reference to the effects of bad teeth on health, the effects of adenoids and nasal obstruction, the reflexes in chorea, the operative treatment of birth palsy, etc. Overlapping has been guarded against, and there is little repetition, but the omissions are such as one does not expect in an ambitious treatise. It is without doubt a valuable work of reference, and no library will be complete without it, but it is doubtful whether it meets the needs of the general practitioner.

### ARCHIVES OF THE MIDDLESEX HOSPITAL.

Archives of the Middlesex Hospital, Volume XII. Edited by a Committee of the Staff of the Hospital. 8vo., pp. 63. Published by Macmillan and Co., Ltd., June 1908. Subscription price, 10s. 6d. per annum.

The amount of clinical material that lies buried in the case-reports of all big hospitals is gigantic. It should be the duty of the archives or reports of each such hospital to sift out the most interesting portions of this clinical material every year, and place it upon record in printed form. We have heard objections to the continued publication of hospital reports, on the grounds that there is already such an immense and increasing bulk of print upon medical subjects, and that the major part of what is published in such reports also appears in the *British Medical Journal*, *The Lancet*, the Proceedings of the Royal Society of Medicine, and so forth. If hospital reports were simply collections of reprints we should agree with this; but many an interesting case can be found in such reports and nowhere else, so that there is a distinct purpose that they serve. Nevertheless, it is a thousand pities that the co-ordination and co-operation of the London hospitals has not advanced sufficiently far to allow of an amalgamation of archives and reports, and of a consequent diminution in the number of separate series of volumes that each medical library is compelled to store upon its shelves. With this proviso we have nothing but praise for the volume before us. It is based upon lines precisely similar to those of other hospital reports, containing contributions upon subjects connected with clinical medicine and surgery, researches carried on in the clinical and pathological laboratories, and in the special departments of the hospital and school. About three such small volumes are issued annually—a mistake, we think, because there is no urgency about any of the contributions, and the three parts might very well be issued annually as a single volume of average size. The subjects dealt with in the one before us are Wertheim's radical abdominal operation for carcinoma of the cervix uteri; diplococcal infection in acute poliomyelitis; lymphangioplasty; diaphragmatic herniæ; and one or two others; and there are nine illustrations of average excellence.

Archives of the Middlesex Hospital, Volume XIII. Pages 208. Illustrations 75.

This, whilst it is the thirteenth volume of the Middlesex Hospital reports, is at the same time the seventh report from the Cancer Research Laboratories, on which account it has a special interest. It is edited for the Cancer Investigation Committee by W. S. Lazarus Barlow, M.D., F.R.C.P., Director of the Cancer Research Laboratories, and it contains papers upon the following subjects: The connective tissues in carcinoma and in certain inflammatory states that precede its onset; secondary malignant conversion of epithelium; lumina in malignant tumours; *x*-ray carcinoma and the conditions which precede its onset; the effects of urinary and biliary calculi, and of certain animal tissues including cancers of various organs, upon photographic plates in the dark; the action of thorium and uranium upon certain ferments; a simple apparatus for drawing from the microscope; a new triple stain; and tabulated synopses of the post-mortem examinations and operations in cases of malignant disease during the year 1907. It is clearly impossible to summarise each paper in a brief review like this. We think the titles give a very fair idea of the nature of the contributions. There are some remarkable discoveries embodied in three separate papers upon the effects of various animal tissues on photographic plates in the dark, and we look forward to more researches upon the subject in a future report. The volume as a whole is of scientific rather than of practical interest, but in view of recent events all will be interested in at least one of the articles, namely, that upon the conditions which precede *x*-ray carcinoma.

### SURGERY.

A System of Syphilis. In six volumes. Edited by D'Arcy Power, M.B., F.R.C.S., and J. Keogh Murphy, M.D., M.Ch., F.R.C.S. Vol. 1. (London, Frowde, Hodder and Stoughton. Pp. 380; plates 53. Price 42s. net.)

About the first volume of the "System of Syphilis" which Messrs. D'Arcy Power and J. Keogh Murphy are editing there is an air of luxury due to a series of excellent plates produced by direct colour photography, which, far from precluding the need for reading the text, is the best incentive to study a work bearing such prominent evidence of careful preparation. "The time has come," say the editors, "when syphilis can be discussed . . . from the standpoint of modern medicine and surgery." This was true even before the discovery of T. pallidum by Schaudinn, to whose memory the six volumes are dedicated. The latter is so recent, however, that Professor Metchnikoff's article on the microbiology of syphilis, interesting as it is, does not display quite the dispassionate detachment desirable in a standard work. Iwan Bloch, too, in a vigorous article on the history of syphilis, has rather too obvious an air of triumph in his vindication of the American origin and Columbian introduction of old-world syphilis. For two articles, those on the general pathology of syphilis, by Dr. Andrewes, and on congenital syphilis, by Dr. Still, we have nothing but unstinted praise. A clear argument and a discriminating choice of words enable the former to present his observations with their full force; the latter has once more shown what good use he has made of his great clinical opportunities. We note especially his important statements that Hutchinsonian teeth are not by any means very common, and that the diagnostic value of Parrot's nodes is doubtful. Mr. Shillitoe, dealing with the primary lesions and early secondaries as seen in the female, has written a very instructive article; it may be hoped that his remarks upon chancre of the cervix uteri will lead to a routine adoption of those thorough methods of investigation which, on the Continent, reveal so much larger a proportion of these cases than in ordinary surgical practice is seen in this country. Colonel Lambkin's article on early lesions in the male, although it bears evidence of wide experience, hardly impresses the reader as do the other contributions to the volume; it is, perhaps, that he was a little cramped by the fear of overlapping articles in subsequent volumes. The writers of the volumes to come will have a clearer field and wider scope; if they do their work as well and as conscientiously as the authors of the present volume, the completed work will be invaluable. If it does anything to advance the campaign against a scourge which in some respects is of more sinister import to the State than tuberculosis, the editors may feel that their labours are amply rewarded; but upon its merits, from the purely professional point of view alone it deserves success, and will, we venture to think, attain it. For its size the book is not heavy, the type is pleasant to read, there is a satisfactory index, and although the price is high we think very few purchasers will regret the expenditure.

### GYNÆCOLOGY AND OBSTETRICS.

A Manual of Midwifery. By T. W. Eden, M.D., F.R.C.P. Obstetric Physician to Out-patients, Charing Cross Hospital; Physician to In-patients, Queen Charlotte's Hospital; and Surgeon to In-patients, Chelsea Hospital for Women. 42 plates and 236 illustrations. Pp. 555. (London: J. and A. Churchill. 1908. Price 10s. 6d. net.)

The fact that only two years have elapsed since the first edition of this work appeared proves that it has been welcomed by students. The new edition contains about fifty additional pages of reading matter, and some subjects are dealt with which were either omitted or very much compressed in the first edition. We confess we should like to see some more common-sense view of the mechanism of labour in vertex presentations, especially with regard to flexion. We feel strongly that the time is ripe for the banishment of the lever theory of flexion. If the lever theory is true it should act equally well in occipito posterior cases, and this we know is frequently not the case. We regard Fothergill's explanation of flexion as a simple theory and one which explains the deflexion which occurs in occipito posterior cases. We are glad to see that the author recommends vaginal plugging without reserve for external accidental hæmorrhage, but think that the original "Dublin method" with cotton-wool plugs which have been boiled and squeezed dry, is preferable to and more efficacious than plugging with gauze. We also note that plugging is recommended for concealed hæmorrhage in the absence of skilled assistance and in surroundings unsuitable for a serious operative procedure such as Cæsarian section. This is at least authoritative, and probably the best plan also. Modern text-books have shown a tendency to beg the question of the treatment of concealed hæmorrhage, and nothing is worse for students than the absence of dogmatic teaching. The final chapters on infant feeding and obstetric operations are very good, and present the subjects in a form which should be of real assistance in practice.

The Edinburgh Stereoscopic Atlas of Obstetrics. Edited by G. F. Barbour Simpson, M.D., F.R.C.P.E., F.R.C.S.E., and Edward Burnet, B.A., M.B., Ch.B., In four sections, each containing 25 subjects with descriptive text. (London. The Caxton Publishing Co. 1908. Price of the four sections, 84s. net.)

The first section of this Atlas deals with the female pelvis, its comparison with that of the male, and all its abnormal and deformed varieties. Each stereoscopic picture is carefully described in a clear and concise manner. The value of stereoscopic vision in demonstrating depth and thickness cannot be over-estimated, and no collection of plates has hitherto been produced which gives such a good idea of the features of the female pelvis and its distortions as the present Atlas. The authors are to be congratulated upon the success of their labours, and their Atlas must prove of the greatest value for teaching purposes in small classes. The stereoscope supplied with the Atlas is very handy, folds up into small compass, and when its intricacies have been mastered forms a very efficient instrument for the purpose for which it was designed.

### MEDICO-LEGAL.

The Law in General Medical Practice.—By Stanley B. Atkinson, M.A., M.B., B.Sc., Barrister-at-Law, Hon. Sec. of the Medico-Legal Society. (London: Henry Frowde, and Hodder and Stoughton, 1908. Pp. 239. Price 7s. 6d. net.)

There has long been wanted a book on this subject, and it is curious that no one has hitherto thought of endeavouring to satisfy that want. Excellent though the idea is, we do not feel that Mr. Atkinson has quite risen to the occasion: his volume is scrappy, and bears the appearance of compilation in undue haste. There is, we should judge, more of the lawyer than the general practitioner about Mr. Atkinson, though we should not consider that in any way a disadvantage if it conduced to clear and consecutive exposition. Not that anyone in medical practice will regret reading through this book; far from it, for here and there he will find counsels of great wisdom on points about which few medical men are well informed. But taken as a whole the work is disappointing, and especially so in the more strictly medical sections. Thus the author sets out to recite the possible causes of abortion, and first of all mentions ectopic gestation, which, he says, "leads inevitably to abortion"; in addition, still with reference to ectopic gestation, we are told that microscopical examination may be necessary to identify the doubtful uterine ejecta as products of conception. After these extraordinary statements we are not surprised to find that the list of causes of abortion omits several of the commonest and most important. Yet Mr. Atkinson is a member of the Central Midwives Board! Nor do we agree that, in considering the expediency of inducing labour or abortion for the safety of a patient with contracted pelvis, "a glance at the size of the husband's head or hat may assist": this, to put it quite bluntly, is mere clap-trap, which should find no place in a book seriously addressed to the medical profession. In the very next sentence it is stated that "abortion . . . . is necessary where the true conjugate is less than two and a half inches." We should not trouble to draw attention to the incomplete and partly inaccurate nature of this statement were it not that the author's position as Secretary of the Medico-Legal Society may cause his words to be regarded as authoritative in courts of law. Surely, too, Mr. Atkinson is incorrect in asserting that "the Notification of Births Act, 1907 . . . . includes every fœtus of more than five months' gestation." The words of Section 1 of the Act, as quoted on official forms, are "after the expiration of the twenty-eighth week of pregnancy."

On defamation, slander, and libel the author is in happier vein; there is much in this section that will interest medical men, and plenty of common sense is interspersed among more strictly legal information. Throughout the book it is evident that many knotty problems in connection with the rights and wrongs of the profession are still unsolved by leading cases; perhaps the best thing for everybody will be that this state of things should long continue, but Mr. Atkinson does good service in pointing out what these problems are, and what views judges or juries might be expected to take on them. But, after all, no one can really tell beforehand what any given jury or judge will decide about unprecedented points in medical jurisprudence.

Mr. Atkinson is certainly extremely happy in his selection of aphorisms and wise saws in illustration of particular points, but this method of exposition has disadvantages when carried to extremes. When we have his own opinions in his own language, the result, if less sparkling, is of more general utility to the harassed practitioner face to face with some legal difficulty. We note with especial pleasure what he has to say on the subject of medical defence societies.

### CLIMATOLOGY.

Mediterranean Winter Resorts. By Eustace Reynolds-Ball, F.R.G.S. Sixth Edition. Pp. 646. (London: Hazell, Watson and Viney. Price 6s.)

Climatology is a branch of therapeutics of which few medical men have any first-hand knowledge, and about which, therefore, they must take on trust the recorded experience of others. But a mere consideration of climatic conditions alone is only a portion of the problem with which one is confronted in any particular case when asked to advise as to a health resort in convalescence or in actual sickness. Besides the suitability of the climate to the special requirements of the patient, there are also most important questions as to the means of reaching it, the nature of the accommodation available, the chances of recreation, amusement, and so forth. The text-book which would inform us fully on all these matters must therefore be ambitious, for it must deal with many matters not in any way connected with medical science, as well as with the conflicting claims advanced on behalf of numerous health resorts by practitioners resident there. It must preserve impartiality and shrink not from home truths at times.

These requirements Mr. Ball's well-known volume fulfils and reconciles very well indeed. The information on railways, steamers, and other methods of communication is thoroughly up to date and reliable. Stock guide-book matter is cut down to the barest minimum, whereas sport, amusements, and legal information, which are of importance to the visitor as opposed to the passing tourist, are fully dealt with. The strictly medical information is, on the whole, good, based as it is in many cases on published papers by recognised authorities in climatology; it is not, of course, infallible, but it bears the appearance of a decent impartiality. In the chapter on sea sickness we notice that the causes of this disease are laid down as effusion of blood to the brain, disturbance of the digestive system, and over-eating or under-eating, a series of statements which does not carry one much further in prophylactic or any other treatment. Taken all round we recommend this book strongly to anyone contemplating for himself or his patient a journey in search of health to any part of the Mediterranean littoral.

Climate. By Robert de Courcy Ward. The Progressive Science Series. (London: Murray. Pp. 370. Price 6s. net.)

If the present age is not richly endowed with men of surpassing genius, it can, at any rate, produce men whose work is both scientific and scholarly. Mr. Ward's book is another example of the effort that is being made towards intelligible heterogeneity. He discusses pleasantly and usefully the influence of climate on man, touching upon questions of diet and disease as noticeable in tropical, temperate, or polar zones. He mentions the widespread idea, particularly in Canada, that climate tends to change; and maintains that such modification is entirely without scientific support. The memory of man, he urges, is essentially treacherous, and the personal equation is far too prominent with the "oldest inhabitant." The book is written in a most popular manner and should be quite intelligible to one who has no expert training or knowledge of the subject.

### MISCELLANEOUS ITEMS.

The Care and Nursing of the Insane. By Percy J. Baily, M.B., C.M. (London, Scientific Press, Ltd. Size $7\frac{1}{4}$ in. by $4\frac{3}{4}$ in. Pp. 270; 22 figures and illustrations. Price 2s. 6d. net.)

This unassuming little book seems to us to be one of the few which can be said with truth to supply a much-felt want. It is a reprint of lectures given by the author, a physician of experience in Asylum work, to the nursing staff at Hanwell Asylum, which have already appeared in the pages of the Nursing Mirror. The subject is divided into five parts, namely: I. Anatomy and Physiology; II. Nursing of the Sick; III. The Germ Theory of Disease and Disinfection; IV. The Symptoms of Disease—Their Observation and Significance; and V. The Mind and its Diseases. In the first four of these five sections is set forth, in a clear and interesting style, all that a nurse will require to know of the subjects for her work in the wards and for the examination for the certificate of the Medico-Psychological Association. The last section is very short and is evidently intended only as an aid to what is being learnt every day in the Asylum wards. A feature which materially adds to the book's usefulness is its index. The book can be highly recommended as a most useful one to all Asylum workers.

Lectures on Babies. By Ralph Vincent, M.D., Physician to the Infants' Hospital, Westminster. (London: Baillière, Tindall, and Cox. 1908. Pp. 114. Price 2s. 6d. net.)

It is not quite obvious at first sight precisely to what nature of audience these lectures were delivered: they seem somewhat advanced for nurses, or indeed anyone but members of the medical profession, and yet they contain much that is common knowledge among fourth-year students. As an exposition in clear form of the views of those who uphold the value of percentage milk mixtures for babies, sick or hale, to whom for any reason their mothers' milk is not being offered, Dr. Vincent's lectures are worthy of high praise. If his advocacy is a little impassioned, it at least is evidently founded on conviction. The near future will decide whether his gospel will constitute the whole law on this matter; but in any case it is worthy of the most serious consideration from every practising physician. One of his most notable—though, we fear, least practicable—suggestions is that of making it "a penal offence for any manufacturer to put forward as a food for infants anything which does not . . . possess the essential qualities of a food to be given to an infant." But this is one of the blessings for which we shall doubtless have to wait until a Ministry of Public Health has been established at Whitehall.

Cancer: Relief of Pain, and Possible Cure. By S. and G. E. Keith. (London: A. and C. Black, 1908. Pp. 155. Price 2s. 6d.).

This volume contains the experience and conclusions of its authors as to the treatment of malignant disease by injections of certain iron, arsenic, and iodine compounds. They are enthusiastic upon the relief of pain, prolongation of life, and occasional cure of apparently hopeless cases by these methods. The value of their treatment can only be determined by independent investigation, and the manner in which their case is presented differs considerably from that of the inventors of various recent remedies now discarded. The illustrative cases, however, prove little, and the preamble contains statements which are, to say the least of it, unjustified by practical experience.

